# Molecular Pathology of Myotonic Dystrophy Type 1 in Iceland

**DOI:** 10.1002/mgg3.70013

**Published:** 2024-10-07

**Authors:** E. G. Hallgrímsdóttir, H. Svansson, V. F. Stefánsdóttir, Ó. Á. Sveinsson, H. Ólafsdóttir, E. Briem, S. Sveinbjörnsdóttir, J. J. Jónsson

**Affiliations:** ^1^ School of Medicine European University Cyprus Nicosia Cyprus; ^2^ Department of Medicine Aarhus University Aarhus Denmark; ^3^ Department of Genetics and Molecular Medicine Landspítali‐National University Hospital of Iceland Reykjavík Iceland; ^4^ Department of Neurology Landspítali‐National University Hospital of Iceland Reykjavík Iceland; ^5^ Faculty of Medicine University of Iceland Reykjavík Iceland; ^6^ MSE Trust, Basildon University Hospital Queen Mary University of Medicine and Dentistry, University of London London UK

**Keywords:** ascertainment, cascade screening, Iceland, myotonic dystrophy, prevalence

## Abstract

**Background:**

Myotonic Dystrophy type 1 (DM1) is an autosomal dominant disease with anticipation due to increased number of CTG repeats in the *DMPK* gene.

**Methods:**

This retrospective, cohort study in Iceland assessed prevalence of DM1, molecular pathology, and patient ascertainment. Data was collected from all major hospitals in Iceland, Medical Director of Health, and independent clinics. Cohort criteria were diagnosis of DM1 on January 1, 2021, or time of death. Population‐based Icelandic Genealogy Database of the Genetical Committee at the University of Iceland was used for genealogy.

**Results:**

In Iceland, 221 individuals, including 19 obligate carriers, had been diagnosed with DM1 of which 144 were alive giving a point prevalence of 39 per 100,000 (four times the world average of 9.3). Genealogy analysis identified 45 first‐degree families. Age‐adjusted prevalence ranged between 11 and 66 per 100,000. Average potential years of life lost were 20.5 per person. Where information was available, 63% of ascertainment was based on family history in cascade testing.

**Conclusion:**

The differences in age‐adjusted prevalence suggest that the overall point prevalence is an underestimation due to underdiagnosis in younger age groups and lethality in oldest age group. Our data supports use of cascade testing to improve DM1 ascertainment.

## Graphical abstract

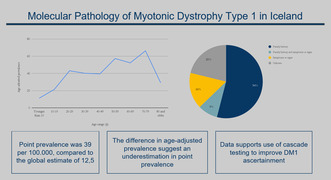

## 1 Introduction

Myotonic dystrophy type 1 (DM1, # 160900) is an incurable inherited degenerative disease with a complex symptomatology (Thornton 2014). It is the most common inherited adult myopathy (Vydra and Rayi 2023). The global prevalence of DM1 differs and has been estimated to be 5–20 per 100,000, with an average global prevalence of 9.3 (Liao et al. 2022; Müller et al. 2021; Harper 2001; Norwood et al. 2009; Siciliano et al. 2001). The prevalence of DM1 in Europe was estimated to be 12.3 per 100,000 (Liao et al. 2022). In 2005 an epidemiological study on the Icelandic DM1 cohort reported a point prevalence of 28.2 per 100,000 (Leifsdóttir et al. 2005). The 2005 prevalence was considerably higher than the 9.6 per 100,000 reported in Iceland in 1968 and suggested a markedly higher prevalence in Iceland compared to the global prevalence (Gudmundsson 1968). However, several regions have reported a higher prevalence, for example, Quebec, Norrbotten Sweden, and New York State with the prevalence of 158.0, 70.0, and 47.6 per 100,000, respectively (Mathieu and Prévost 2012; Olofsson et al. 1988; Johnson et al. 2021). The high prevalence in Quebec and Norrbotten is presumably due to a founder effect (Thornton 2014; Leifsdóttir et al. 2005). This might also be the case in Iceland, where founder effects are common (Jensson et al. 2023).

The genetic pathology in DM1 is trinucleotide instability in the dystrophia myotonica protein kinase (*DMPK*, * 605377) gene (Brook et al. 1992). An expansion of more than 49 tandem CTG repeats in the 3′ untranslated region of the *DMPK* gene on chromosome 19 (NM_004409.4(DMPK):c.*224_226CTG(50‐?)) is considered pathological. The consequences of the CTG expansion are multisystemic, resulting in broad symptomatology of variable severity (Ashizawa et al. 2000). Consequently, diagnosing individuals based on symptoms or signs can be difficult.

The inheritance of DM1 is autosomal dominant with anticipation (Harper et al. 1992). Three clinical forms of DM1 have been categorized into congenital, classic, and mild, based on age of onset and symptoms. The classic form has been further classified into three sub‐forms depending on age of onset: childhood, juvenile, and adult (Gutiérrez Gutiérrez et al. 2020). The disease severity is directly correlated with the number of increased CTG repeats (Yum, Wang, and Kalsotra 2017). The congenital form is the most severe form of DM1 and is sometimes fatal in the newborn period. The average lifespan of individuals with the congenital and classical forms of DM1 is reduced. In contrast, patients with the mild form of DM1 have a normal lifespan with limited or no detectable signs (Thornton 2014).

DM1 is primarily diagnosed with genetic testing. Electromyography (EMG) has less sensitivity and specificity (Savić Pavićević et al. 2013; Barnes et al. 1994). Importantly, genetic testing is the only way of diagnosing asymptomatic relatives in cascade testing (Meola and Cardani 2015). Cascade testing is an effective way of identifying high‐risk individuals and enabling surveillance. It is a systematic approach of informing relevant family members about their own risk of developing the disease following the identification of a pathogenic variant in an index patient. Involving the proband in the process is the most common method. However, the uptake seems to be higher when healthcare professionals inform other family members (Menko et al. 2019; Srinivasan et al. 2020).

Clinical genetic testing has limitations, the most important being that the range of number of repeats in expansions is not always reported and that the number of repeats can vary with age and differ between tissues (Addis et al. 2012; Ballester‐Lopez et al. 2020).

The importance of genetic testing was demonstrated in a study done in 2021, where an unbiased screening of newborn blood samples in New York state yielded a prevalence 47.6 per 100,000 (Johnson et al. 2021). This suggests that DM1 is significantly underdiagnosed.

This paper describes a whole population retrospective cohort study on the Icelandic population. The goal of this study was to reassess the prevalence of DM1, describe the molecular pathology, and identify factors leading to patient ascertainment. In addition, we examined the relationship between repeat number and life expectancy in an effort to define a patient cohort to study the effect of an active surveillance program and the effect of therapy.

## 2 Materials and Methods

### 2.1 Ethical Compliance

Study was approved by the National Bioethics Committee (VSN‐21‐107).

### 2.2 Data

All pre‐existing data was collected and accessed from the following institutions: Landspítali‐National Hospital of Iceland, Akureyri Hospital, Medical Director of Health of Iceland, and independent clinics in Iceland. The following ICD numbers were used to identify patients. ICD9: 359.0, 3591.1, 359.2, 359.8, and 359.9 and ICD10: G71.0, G71.1, G71.2, G71.3, G71.8, G71.9 and Z82.0. Results were collected from all clinical testing of *DMPK* gene at Landspítali‐National University Hospital of Iceland, the only laboratory testing center in Iceland for *DMPK* genetic repeat expansions. Clinical testing was done with PCR (*N* = 54), triple‐repeat primed PCR (*N* = 34), and Southern blotting (*N* = 49). Information regarding age, gender, and history of disease were obtained through medical records and encrypted for further analysis.

Diagnostic criteria for the patient cohort were a pathologic result from *DMPK* genetic testing; a positive EMG test for DM1; clinical diagnosis of DM1 or obligate carrier in the pedigree. The cohort was further limited by an Icelandic residency, according to the National Registry in Iceland (Þjóðskra 2021) on January 1, 2021, or at the time of death.

Genealogical analysis on the cohort was done via the population‐based Icelandic Genealogy Database of the Genetical Committee at the University of Iceland. This multigenerational population genealogy database includes individuals born after 1845. Pedigree analysis was conducted with CJC Pedigree Software (Chapman 2016; Tulinius 2011).

### 2.3 Calculations

Total number of individuals living in Iceland on January 1, 2021 (368,792) and the number of individuals according to sex and age group were collected from Statistics Iceland (Hagstofa Íslands 2021). The national sex ratio (male: female) was 1.05:1. All relevant clinical data collected on individuals with DM1 was until January 1, 2021. Chi‐Square calculations were done using an online tool (Social Science Statistics 2021). Excel was used to calculate the average and median of different values. Potential years of life lost (PYLL) was calculated with the reference age of 75 (OECD 2021). Mann–Whitney *U* test was done with Python (Python Software Foundation 2022; Virtanen et al. 2020).

Molecular genetic testing was based on the reference sequence (NM_004409.4[DMPK]:c.*224_226CTG[50‐?]). The results from clinical genetic testing were classified either as normal, less than 50 repeats, or as pathological, if the CTG repeats were 50 or higher (Ashizawa et al. 2000). If the results were pathological, the results were further classified as (i) a specific number, (ii) a range, or (iii) an open‐ended answer. If an individual received a range for their CTG repeat number, the midpoint of the range was used.

## 3 Results

In total, 221 individuals had been diagnosed with DM1 in Iceland, of whom 144 were alive at the time of the study. The point prevalence of DM1 on 1 January 2021 was 39 per 100,000. Of those diagnosed with DM1, 19 were obligate carriers of the disease. *Genealogical analysis of individuals diagnosed with DM1 in Iceland and their families identified 45 first degree families*. First degree families comprised either 7 single affected individuals with no identified genealogy relationship with other patients or *where all individuals in a family had first degree relationships with at least one other affected individual*. The two largest families contained 25 and 23 individuals, respectively. *When criteria were relaxed to include third‐degree relatedness, two families merged*, *yielding 44 remaining families with third‐degree relatedness. The identified families appeared not to be clustered and no sector distribution of families in the population suggestive of founder effect was identified*.

**FIGURE 1:**
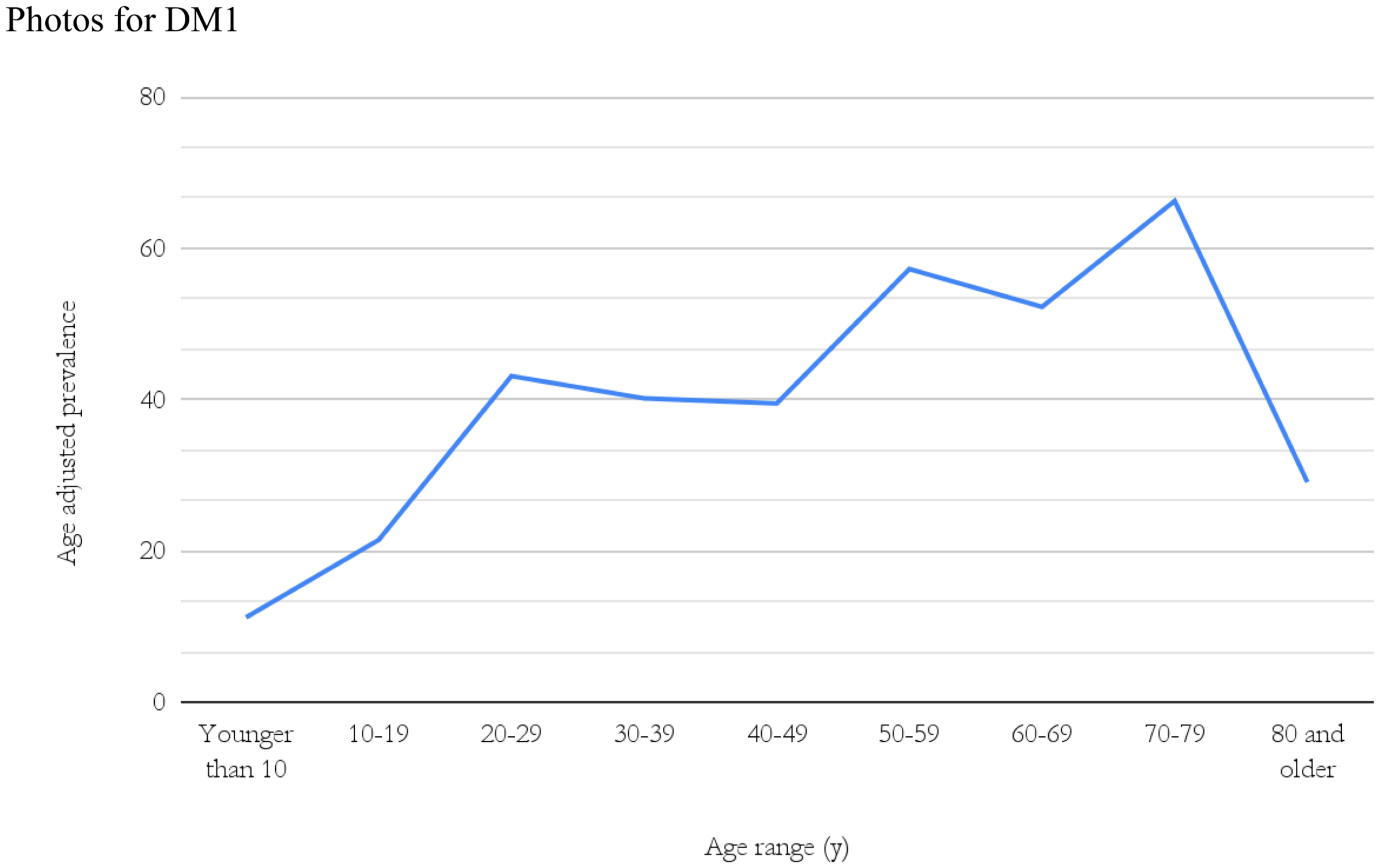
Age‐adjusted prevalence of DM1 in Iceland January 1, 2021.

Of the 144 individuals alive, 79 were male and 65 were female (sex ratio 1.2:1). No statistically significant difference was observed between the cohort sex ratio and that of the general Icelandic population (*p* = 0.4) (OECD 2021). The average age of males was 47 years (median 50 years) and 42 for females (median 43 years). Of the 221 individuals diagnosed with DM1, 159 (72%) had a reported age at their diagnosis. The average age of males at diagnosis was 37 years and for females 32 years. The age‐adjusted prevalence varied between age groups (Figure 1).

**FIGURE 2:**
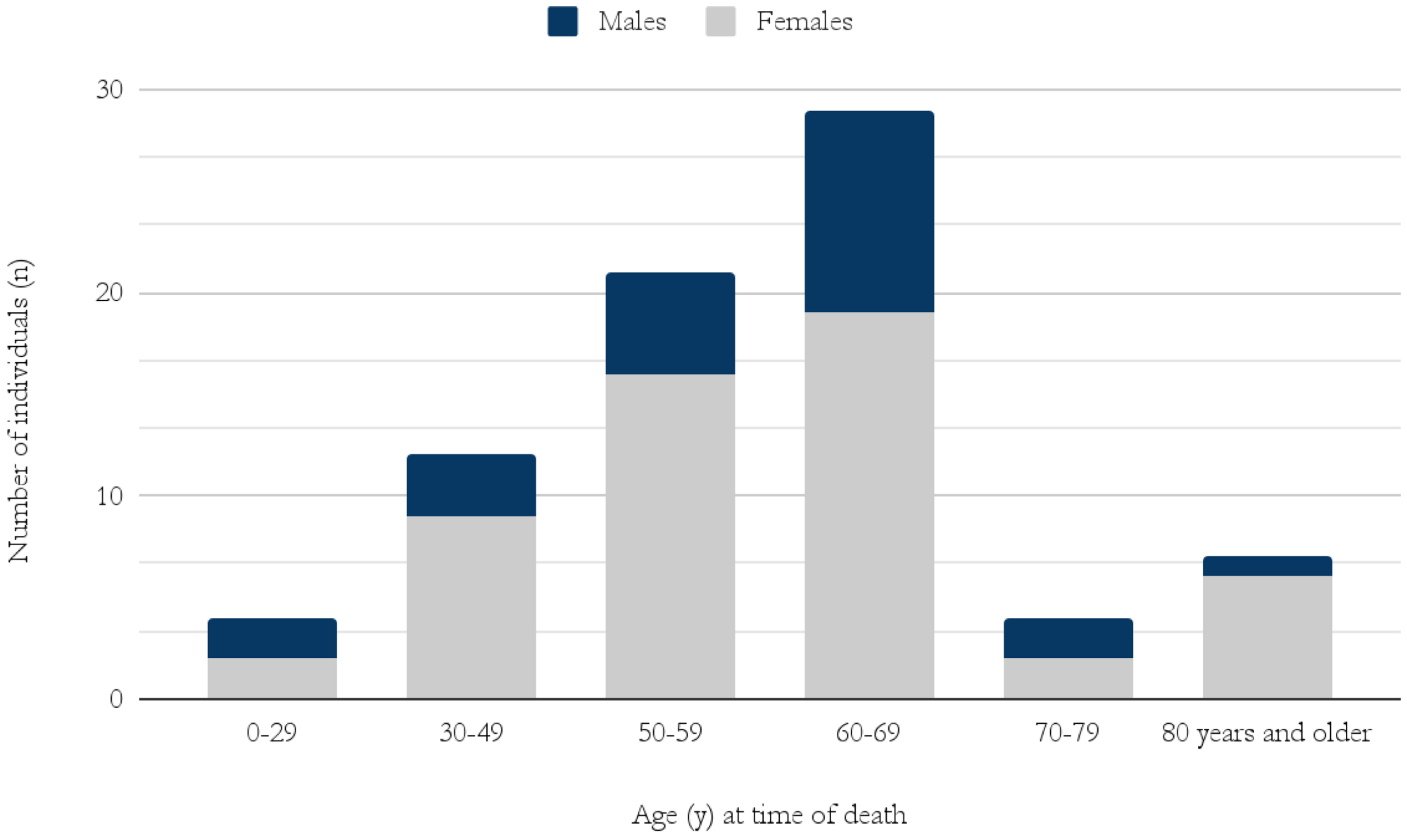
Age at the time of death of males and females diagnosed with DM1 in Iceland.

Of the cohort, 77 were deceased (23 males and 54 females). The average age at the time of death was 57 years for males (median 61 years) and 58 years for females (median 60 years) (Figure 2). A Mann–Whitney *U* test was conducted on the cohort, where the relationship between gender and average age of death was assessed. No statistically significant difference was observed (*p* = 0.6) (Python Software Foundation 2022; Virtanen et al. 2020). The average PYLL was 20.5 years per person. Of the 77 deceased, 20 (29%) had a known cause of death (OECD 2021). Of those, 5 had the reported cause of death as cardiac arrest, and 13 had respiratory failure.

**FIGURE 3:**
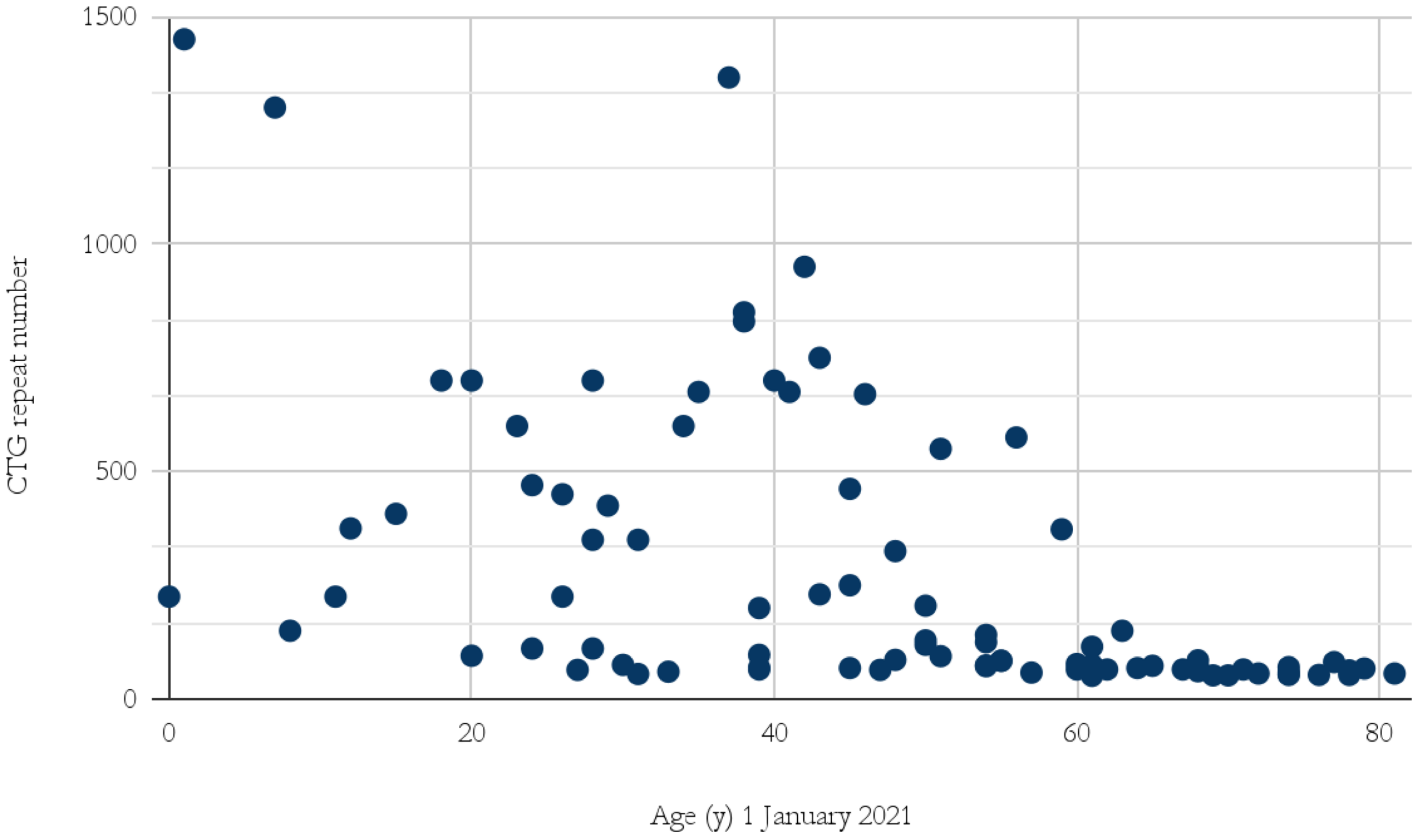
Number of CTG repeats and age of those alive January 1, 2021, in Iceland. Individuals who received open‐ended answers could not be included in this chart.

Of the cohort, 137 (62%) had undergone genetic testing, of which 73 were male (63% of all diagnosed males) and 64 were female (61% of all diagnosed females). PCR 39% (*n* = 54), TP PCR 25% (*n* = 34) and Southern blotting 36% (*n* = 49). The number of CTG repeats in individuals aged 60 years and older never exceeded 200. Individuals with more than a thousand CTG repeats were all younger than 40 years (Figure 3).

**FIGURE 4:**
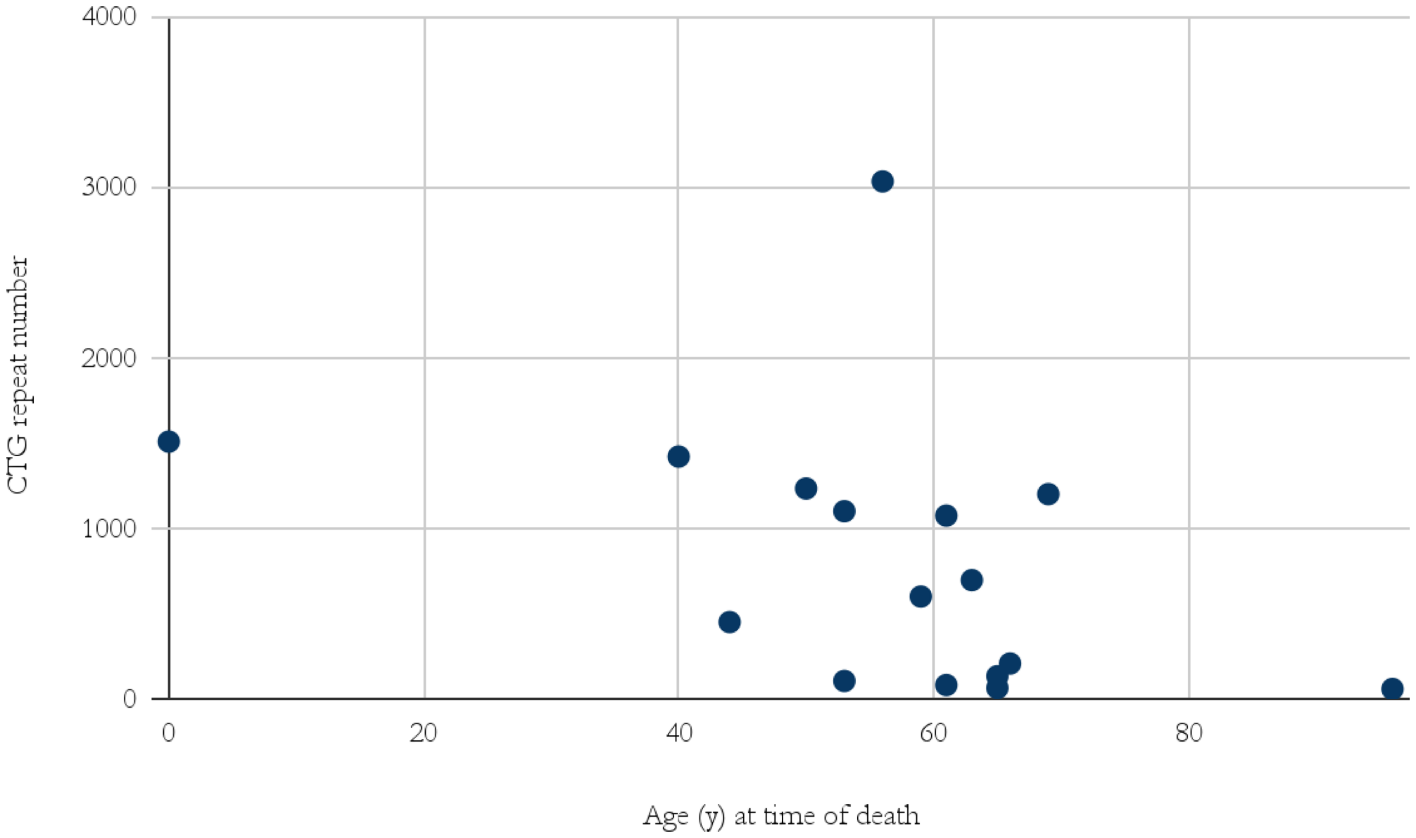
Age at time of death of individuals diagnosed with DM1 and their CTG repeat number.

The majority of individuals diagnosed with DM1 passed away between the ages of 40 and 70 years (Figure 4).

Of those who underwent genetic testing, 121 individuals also received the repeat length of the non‐expanded allele. The most common repeat length of the non‐expanded allele was 5 (36%) and between 11 and 15 (46%).

**FIGURE 5:**
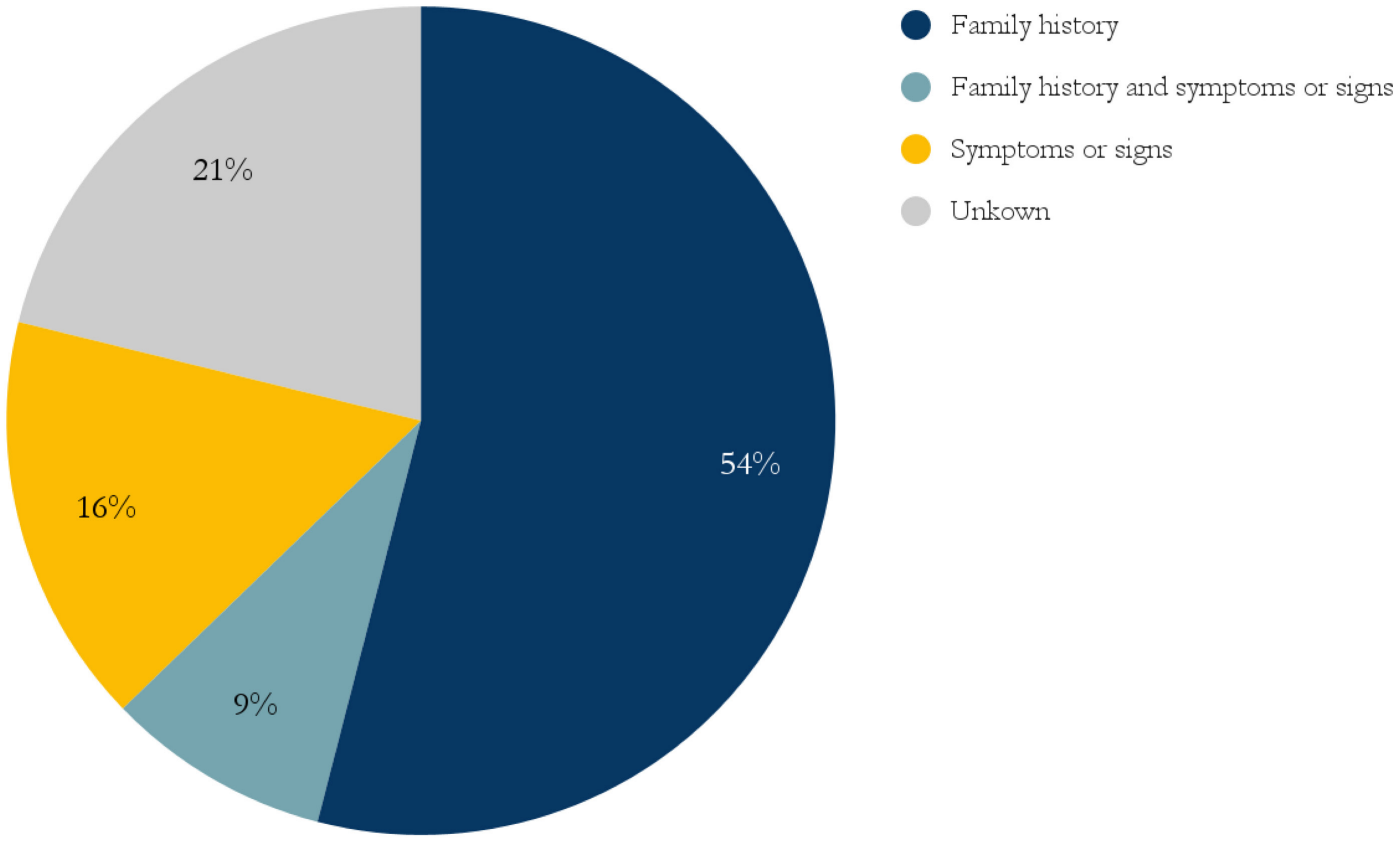
Indications for genetic testing.

Of the cohort, 108 (79%) had a documented indication(s) for genetic testing (Figure 5). Family history was the most common indication for testing (63%) either alone (54%) or with symptoms and signs also listed (9%).

**FIGURE 6:**
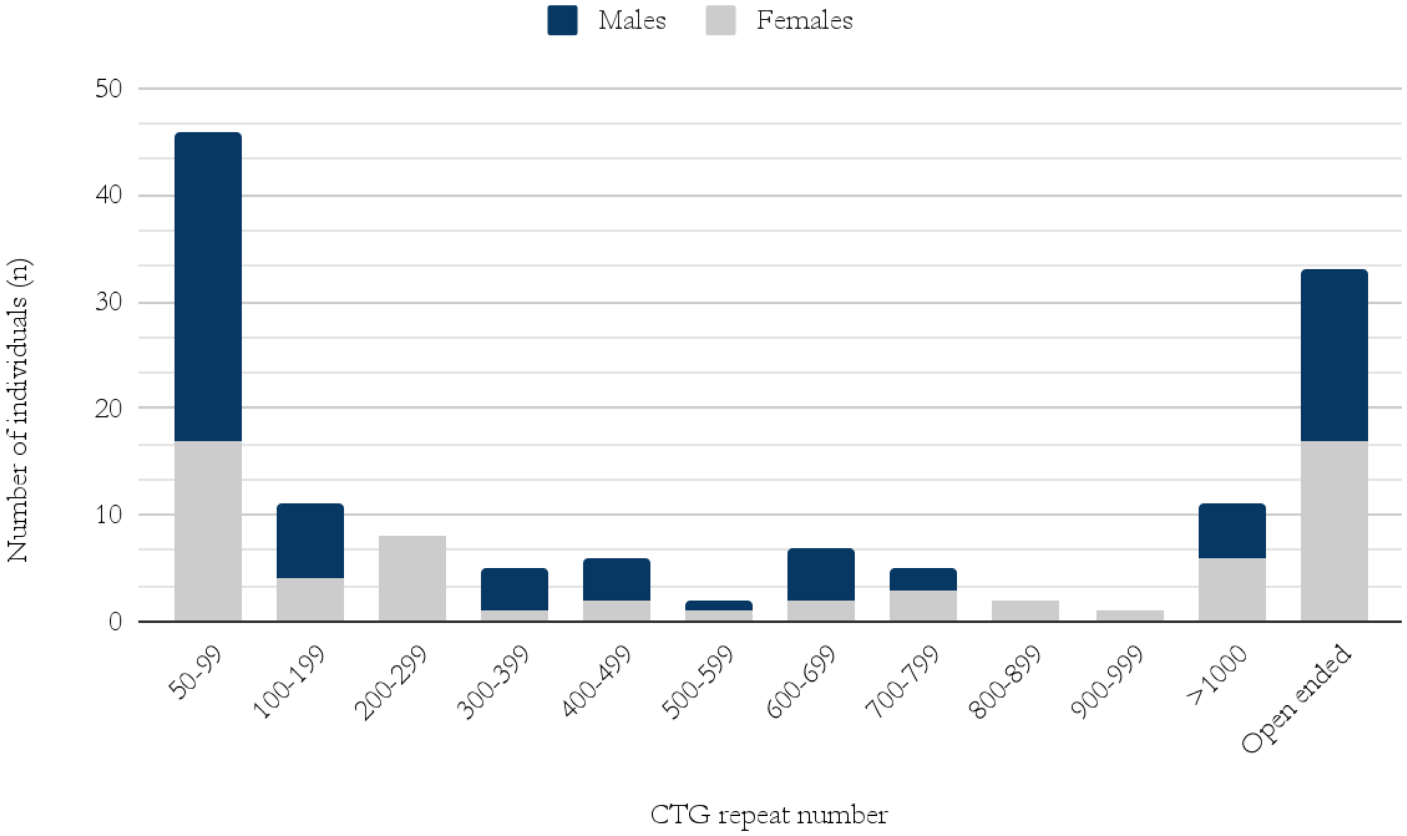
Number of CTG repeats in the pathological allele in males and females diagnosed with DM1 in Iceland.

In total, 104 individuals received either a specific number or a range on *DMPK* genetic testing and 33 individuals received an open‐ended result (Figure 6).

**FIGURE 7:**
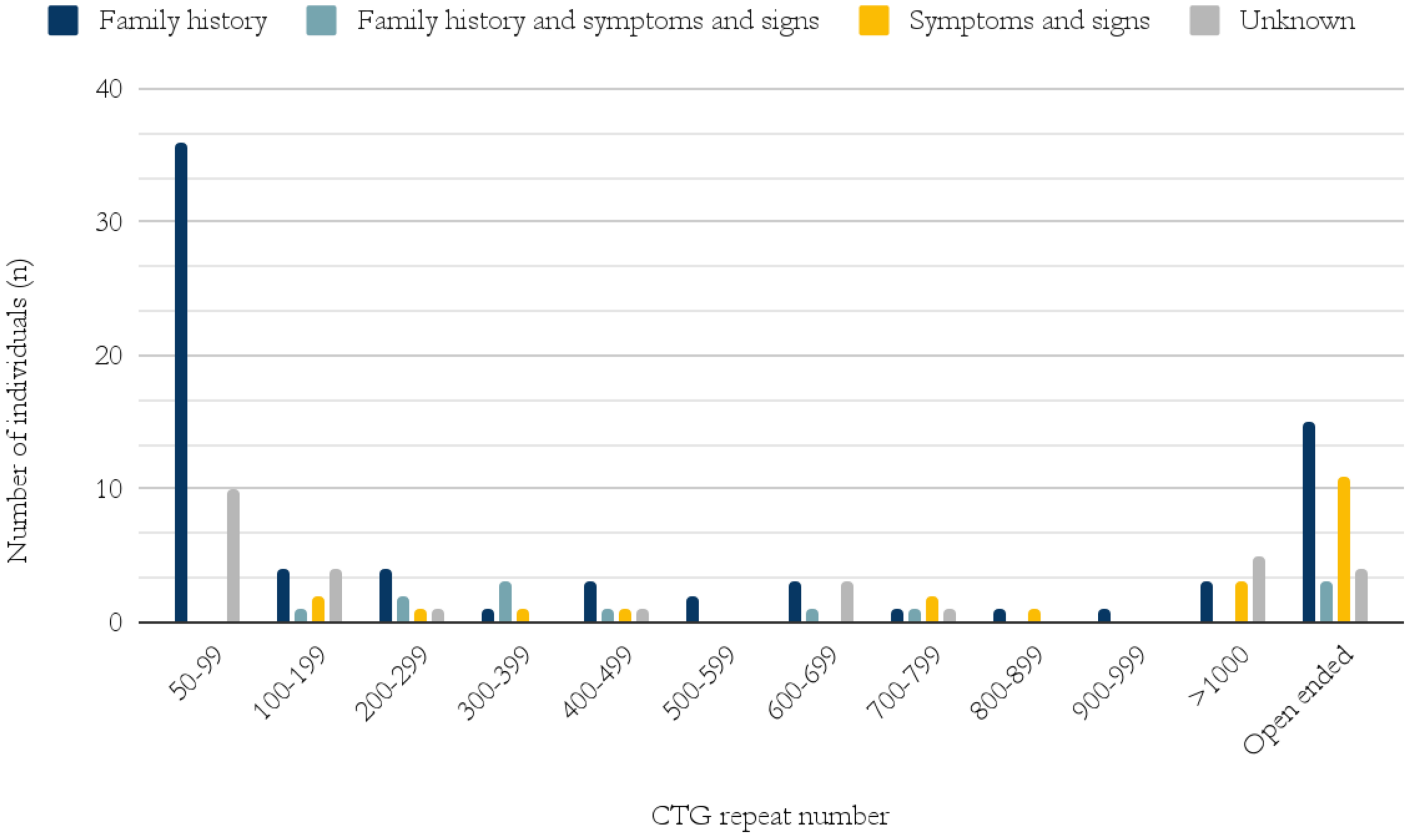
Indication(s) for diagnostic genetic testing and CTG repeat number.

All individuals with less than 100 CTG repeats, and where the indication for diagnosis was documented, were tested due to a family history of the disease (Figure 7).

## 4 Discussion and Conclusion

This retrospective cohort study revealed a point prevalence of 39 per 100,000 in Iceland on 1 January 2021 which is four times the world average estimate (Liao et al. 2022). The current study attempted to identify all DM1 patients in Iceland through a comprehensive analysis of preexisting health records and genealogy analysis. Since the cohort was limited to patients already diagnosed with DM1, the reported figures are a minimum prevalence. The strength of the study was that it was a nationwide study, built also on data on genetic testing from the Dept. of Genetics and Molecular Medicine at Landspítali—National University Hospital of Iceland which has been operational since 2002. It is the only formal unit in Iceland offering genetic testing and counseling for DM1.

It is difficult to estimate how many undiagnosed DM1 patients remain in Iceland. An interesting insight can be found in the data on age‐adjusted prevalence. In theory, all age categories should have the same prevalence based on the causative pathologic allele frequency. Thus, the highest prevalence category, for example, *n* = 66 DM1 individuals per 100,000 (Figure 1) in the 70–79 age group, represents the best estimate and the lower prevalence in other age groups reflects both underdiagnoses and death due to disease. This assumption suggests considerable underdiagnosis in the younger age groups. We found that the lowest age‐adjusted prevalence was in the age group 0–9 years with 11 per 100,000. The low prevalence in this age group presumably reflects in part lethality in patients with the congenital form (Figure 4), but mostly because asymptomatic individuals younger than 18 are not genetically tested, regardless of family history. Taking these confounding factors into account the true prevalence of DM1 in Iceland could be comparable to the result of 47.6 per 100,000 from the unbiased genetic screening study of newborns in New York (Johnson et al. 2021).

As a previous study had been done on this population (Leifsdóttir 2005), it served as a point of reference to our results. In comparison there was a 39% increase in the reported prevalence. Furthermore, our results defined 44 DM1 families when viewed through third‐degree relatives in contrast to the 10 families identified by Leifsdóttir et al. (2005). The increased number of affected families is thought to represent increased ascertainment of patients. At the same time the families have many relatives that had not been examined, yet some are affected contributing to underrepresentation in our data. The high prevalence of DM1 in Iceland may be due to founder effects, as it is common in other diseases in Iceland (Jensson et al. 2023). However, analysis of the Icelandic multigenerational database did not identify patterns suggestive of a founder gene. Such a gene may be difficult to identify given anticipation. The distribution of DM1 patient characteristics in Iceland, such as genotype, phenotype, life expectancy, and sex ratio, was in accordance with what has generally been observed (Gutiérrez Gutiérrez et al. 2020). Available clinical data differed greatly between patients in this retrospective study based on preexisting medical records. However, from the collected data and from experience (unpublished) the clinical forms and features of the Icelandic DM1 patients were in accordance with repeat number and international literature (Vydra and Rayi 2023). We presume the difference in prevalence is due to improved ascertainment of DM1 cases with cascade testing, rather than increased incidence of the disease given the short time period between the studies (see below).

The average age of those alive was found to be 47 years for males and 42 years for females. The severity of DM1 is clear when observing the average age at the time of death, which was 57 years and 58 years for males and females, respectively. This is considerably lower than the average life expectancy of an Icelander, being 81 years and 84 years for males and females, respectively (Hagstofa Íslands 2021). This demonstrates the severity of the disease. Previous studies on DM1 have found that males have a decreased life expectancy compared to females (Dogan et al. 2016). However, there was no statistical difference in life expectancy between genders in our cohort. This could be due to the few number of deceased males identified. PYLL was calculated from the reference age of 75 years. The years of life lost of the cohort is 20.5 years per person, this loss is more profound if the longevity of the Icelandic population is considered.

Notably, the majority of patients that were deceased at the time of the study were females. We think that this represents bias when observing historical data, as both the current study and the literature have found no difference in sex ratio. The average age of diagnosis in DM1 patients is 37 years and 32 years for males and females, respectively. Earlier diagnosis, a multidisciplinary follow‐up and patient education could improve quality of life and possibly increase life expectancy.

Of those diagnosed with DM1 in Iceland, 62% had undergone genetic testing. The most common non‐pathogenic alleles were 5 and between 11 and 15 CTG repeat numbers which is similar as in other studies (Amenabar et al. 2009; Tishkoff et al. 1998). The most common pathological alleles were CTG repeat numbers between 50 and 100 CTG repeats (34%). All individuals with these repeat numbers were tested for DM1 due to a family history of the disease. This is consistent with mild or no symptoms associated with such alleles. No individual over 60 years of age had a CTG repeat number higher than 200. In comparison, all individuals with an observed CTG repeat number over 1000 were younger than 50 years old (Figure 3). This demonstrates the close relationship between repeat number and life expectancy in the Icelandic DM1 population. However, five of those deceased reached 50 years of age and had a CTG repeat number over 1000, the oldest dying at 69 years of age. Thus historically there is prospect of DM1 patients with CTG repeat numbers over 1000 living past 50.

Of those with a known indication for diagnosis, 78% were diagnosed with genetic testing based on family history alone, and not by clinical presentation. In some instances, there could be lack of initiative of those with DM1 to seek medical help, as apathy is a known symptom of the disease (Miller et al. 2021). As DM1 is characterized by heterogeneous symptomatology, diagnosing the disease based on symptoms alone is difficult especially if symptoms are absent or mild.

Our study demonstrates the importance of cascade testing based on family history in diagnosing DM1 patients (Figures 5 and 7). The authors are not aware of scientific publications describing the percentage of patients that are diagnosed with DM1 due to family history/cascade testing as opposed to clinical manifestations. Presumably, the percentage varies widely between countries.

The results fit well with ongoing discussions in the literature regarding cascade testing in autosomal dominant diseases (Roberts et al. 2018; Kellogg et al. 2020). Guidelines differ on whether healthcare professionals should contact family members directly, following a positive outcome from genetic testing of an index patient. There are ethical and practical considerations in cascade testing on how at‐risk family members should be approached and who should inform them (Clarke and Wallgren‐Pettersson 2019). In Iceland, the current practice is that patients should first inform their relatives. This can be highly problematic in DM1 as the information is complex and the newly diagnosed patient might be apathetic. Given these concerns, and the life‐threatening severity of DM1 (Figures 2, 3, 4), it is reasonable to enhance cascade testing. This could include active planning with the patient on how relatives should be contacted and possible direct contact of relatives by genetic professionals.

As there is no known disease‐modifying treatment for DM1, interventions are limited to alleviation of manifestations. These include treatments for arrhythmia, sleep apnea, and diabetes as well as physical therapy. The effects of these treatments are difficult to document. We have collected data which can serve as historical controls on life expectancy. The current patient population could be used to study the effects of surveillance and treatments on life expectancy as well as the population molecular genetics of DM1.

This retrospective study was based on measuring number of repeats in pathological alleles. It would be interesting to use this cohort in the future to study the relationship between number of repeats, *DMPK* repeat sequencing, sex of transmitting parent, and risk of expansion/reduction.

## Author Contributions

The authors contributed significantly to the research study and read the manuscript. E.G.H., H.S., medical students, and J.J.J. medical geneticist planned the study and wrote the manuscript with input from other authors. V.F.S. genetic counselor and E.B. head laboratory manager contributed to data collection. Ó.Á.S. neurologist input on neuroepidemiology. S.S. neurologist data contribution and insight into previous studies on myotonic dystrophy in Iceland. H.Ó. genealogist did the genealogical analysis.

## Conflicts of Interest

The authors declare no conflicts of interest.

## Data Availability

Anonymous data used in this paper to support the results is available upon request from the corresponding author.

## References

[mgg370013-bib-0001] Addis, M. , M. Serrenti , C. Melone , M. Cau , and M. A. Melis . 2012. “Triplet‐Primed PCR is More Sensitive Than Southern Blotting‐Long PCR for Diagnosis of Myotonic Dystrophy Type 1.” Genetic Testing and Molecular Biomarkers 16, no. 12: 1428–1431. 10.1089/gtmb.2012.0218. 23030650

[mgg370013-bib-0002] Amenabar, F. , H. Jorquera , M. Acuña , and L. Cifuentes . 2009. “CTG Repeats at the Myotonic Protein Kinase Gene in a Healthy Chilean Population Sample.” Acta Neurologica Scandinavica 119, no. 5: 321–324. 10.1111/j.1600-0404.2008.01096.x. 18798829

[mgg370013-bib-0003] Ashizawa, T. , I. Gonzales , N. Ohsawa , et al. 2000. “New Nomenclature and DNA Testing Guidelines for Myotonic Dystrophy Type 1 (DM1). The International Myotonic Dystrophy Consortium (IDMC).” Neurology 54, no. 6: 1218–1221. 10.1212/wnl.54.6.1218. 10746587

[mgg370013-bib-0004] Ballester‐Lopez, A. , E. Koehorst , I. Linares‐Pardo , et al. 2020. “Preliminary Findings on CTG Expansion Determination in Different Tissues From Patients With Myotonic Dystrophy Type 1.” Genes 11, no. 11: 1321. 10.3390/genes11111321. 33171734 PMC7695006

[mgg370013-bib-0005] Barnes, P. R. , D. Hilton‐Jones , G. Norbury , A. Roberts , and S. M. Huson . 1994. “Incorrect Diagnosis of Myotonic Dystrophy and Its Potential Consequences Revealed by Subsequent Direct Genetic Analysis.” Journal of Neurology, Neurosurgery, and Psychiatry 57, no. 5: 662. 10.1136/jnnp.57.5.662. PMC1072951 8201360

[mgg370013-bib-0006] Brook, J. D. , M. E. McCurrach , H. G. Harley , et al. 1992. “Molecular Basis of Myotonic Dystrophy: Expansion of a Trinucleotide (CTG) Repeat at the 3′ End of a Transcript Encoding a Protein Kinase Family Member.” Cell 68, no. 4: 799–808. 10.1016/0092-8674(92)90154-5. 1310900

[mgg370013-bib-0007] Chapman, C. 2016. “Clinical Pedigree.” CJC Pedigree Software Ltd Accessed January 15, 2024. https://clinicalpedigree.com/index.html.

[mgg370013-bib-0008] Clarke, A. J. , and C. Wallgren‐Pettersson . 2019. “Ethics in Genetic Counselling.” Journal of Community Genetics 10, no. 1: 3–33. 10.1007/s12687-018-0371-7. 29949066 PMC6325035

[mgg370013-bib-0009] Dogan, C. , M. De Antonio , D. Hamroun , et al. 2016. “Gender as a Modifying Factor Influencing Myotonic Dystrophy Type 1 Phenotype Severity and Mortality: A Nationwide Multiple Databases Cross‐Sectional Observational Study.” PLoS One 11, no. 2: e0148264. 10.1371/journal.pone.0148264. 26849574 PMC4744025

[mgg370013-bib-0010] Gudmundsson, K. R. 1968. “The Prevalence of Some Neurological Diseases in Iceland.” Acta Neurologica Scandinavica 44, no. 1: 57–69. 10.1111/j.1600-0404.1968.tb07443.x. 5706984

[mgg370013-bib-0011] Gutiérrez Gutiérrez, G. , J. Díaz‐Manera , M. Almendrote , et al. 2020. “Clinical Guide for the Diagnosis and Follow‐Up of Myotonic Dystrophy Type 1, MD1 or Steinert's Disease. [Guía clínica para el diagnóstico y seguimiento de la distrofia miotónica tipo 1, DM1 o enfermedad de Steinert].” Neurología 35, no. 3: 185–206. 10.1016/j.nrl.2019.01.001. 31003788

[mgg370013-bib-0012] Harper, P. S. 2001. Myotonic Dystrophy, 3rd ed. London: WB Saunders.

[mgg370013-bib-0013] Harper, P. S. , H. G. Harley , W. Reardon , and D. J. Shaw . 1992. “Anticipation in Myotonic Dystrophy: New Light on an Old Problem.” American Journal of Human Genetics 51, no. 1: 10–16. 1609789 PMC1682874

[mgg370013-bib-0014] Jensson, B. O. , G. A. Arnadottir , H. Katrinardottir , et al. 2023. “Actionable Genotypes and Their Association With Life Span in Iceland.” New England Journal of Medicine 389, no. 19: 1741–1752. 10.1056/NEJMoa2300792. 37937776

[mgg370013-bib-0015] Johnson, N. E. , R. J. Butterfield , K. Mayne , et al. 2021. “Population‐Based Prevalence of Myotonic Dystrophy Type 1 Using Genetic Analysis of Statewide Blood Screening Program.” Neurology 96, no. 7: e1045–e1053. 10.1212/WNL.0000000000011425. 33472919 PMC8055332

[mgg370013-bib-0016] Kellogg, G. , B. Thorsson , Y. Cai , et al. 2020. “Molecular Screening of Familial Hypercholesterolemia in Icelanders.” Scandinavian Journal of Clinical and Laboratory Investigation 80, no. 6: 508–514. 10.1080/00365513.2020.1795919. 32706999

[mgg370013-bib-0017] Leifsdóttir, G. , J. E. Benedikz , G. Jóhannesson , J. J. Jónsson , and S. Sveinbjörnsdóttir . 2005. “Spennuvisnun (Dystrophia Myotonica): Almennt Yfirlit og Algengi á Islandi [Prevalence of Myotonic Dystrophy in Iceland].” Læknablađiđ 91, no. 11: 829–834. 16264243

[mgg370013-bib-0018] Liao, Q. , Y. Zhang , J. He , and K. Huang . 2022. “Global Prevalence of Myotonic Dystrophy: An Updated Systematic Review and Meta‐Analysis.” Neuroepidemiology 56, no. 3: 163–173. 10.1159/000524734. 35483324

[mgg370013-bib-0019] Mathieu, J. , and C. Prévost . 2012. “Epidemiological Surveillance of Myotonic Dystrophy Type 1: A 25‐Year Population‐Based Study.” Neuromuscular Disorders: NMD 22, no. 11: 974–979. 10.1016/j.nmd.2012.05.017. 22858159

[mgg370013-bib-0020] Menko, F. H. , J. A. Ter Stege , L. E. van der Kolk , et al. 2019. “The Uptake of Presymptomatic Genetic Testing in Hereditary Breast‐Ovarian Cancer and Lynch Syndrome: A Systematic Review of the Literature and Implications for Clinical Practice.” Familial Cancer 18, no. 1: 127–135. 10.1007/s10689-018-0089-z. 29846880

[mgg370013-bib-0021] Meola, G. , and R. Cardani . 2015. “Myotonic Dystrophies: An Update on Clinical Aspects, Genetic, Pathology, and Molecular Pathomechanisms.” Biochimica et Biophysica Acta 1852, no. 4: 594–606. 10.1016/j.bbadis.2014.05.019. 24882752

[mgg370013-bib-0022] Miller, J. N. , A. Kruger , D. J. Moser , et al. 2021. “Cognitive Deficits, Apathy, and Hypersomnolence Represent the Core Brain Symptoms of Adult‐Onset Myotonic Dystrophy Type 1.” Frontiers in Neurology 12: 700796. 10.3389/fneur.2021.700796. 34276551 PMC8280288

[mgg370013-bib-0023] Müller, K. I. , M. V. Ghelue , I. Lund , C. Jonsrud , and K. A. Arntzen . 2021. “The Prevalence of Hereditary Neuromuscular Disorders in Northern Norway.” Brain and Behavior: A Cognitive Neuroscience Perspective 11, no. 1: e01948. 10.1002/brb3.1948. PMC7821572 33185984

[mgg370013-bib-0024] Norwood, F. L. , C. Harling , P. F. Chinnery , M. Eagle , K. Bushby , and V. Straub . 2009. “Prevalence of Genetic Muscle Disease in Northern England: In‐Depth Analysis of a Muscle Clinic Population.” Brain: A Journal of Neurology 132, no. Pt 11: 3175–3186. 10.1093/brain/awp236. 19767415 PMC4038491

[mgg370013-bib-0025] OECD . 2021. “Potential Life Lost.” June 5, 2023. https://data.oecd.org/healthstat/potential‐years‐of‐life‐lost.htm.

[mgg370013-bib-0026] Olofsson, B. O. , H. Forsberg , S. Andersson , P. Bjerle , A. Henriksson , and I. Wedin . 1988. “Electrocardiographic Findings in Myotonic Dystrophy.” British Heart Journal 59, no. 1: 47–52. 10.1136/hrt.59.1.47. 3342149 PMC1277071

[mgg370013-bib-0027] Python Software Foundation . 2022. “Python Language Reference.” version 3.10 Accessed June 5, 2023. https://www.python.org/.

[mgg370013-bib-0028] Roberts, M. C. , W. D. Dotson , C. S. DeVore , et al. 2018. “Delivery of Cascade Screening for Hereditary Conditions: A Scoping Review of the Literature.” Health Affairs (Project Hope) 37, no. 5: 801–808. 10.1377/hlthaff.2017.1630. 29733730 PMC11022644

[mgg370013-bib-0029] Savić Pavićević, D. , J. Miladinović , M. Brkušanin , et al. 2013. “Molecular Genetics and Genetic Testing in Myotonic Dystrophy Type 1.” BioMed Research International 2013: 391821. 10.1155/2013/391821. 23586035 PMC3613064

[mgg370013-bib-0030] Siciliano, G. , M. Manca , M. Gennarelli , et al. 2001. “Epidemiology of Myotonic Dystrophy in Italy: Re‐Apprisal After Genetic Diagnosis.” Clinical Genetics 59, no. 5: 344–349. 10.1034/j.1399-0004.2001.590508.x. 11359466

[mgg370013-bib-0031] Social Science Statistics . 2021. “Chi Square Calculations.” Accessed June 5, 2023. https://www.socscistatistics.com/tests/chisquare2/default2.aspx.

[mgg370013-bib-0032] Srinivasan, S. , N. Y. Won , W. D. Dotson , S. T. Wright , and M. C. Roberts . 2020. “Barriers and Facilitators for Cascade Testing in Genetic Conditions: A Systematic Review.” European Journal of Human Genetics: EJHG 28, no. 12: 1631–1644. 10.1038/s41431-020-00725-5. 32948847 PMC7784694

[mgg370013-bib-0033] Íslands, H. , and Statistics Iceland . 2021. “Mannfjöldi.” Accessed June 5, 2023. https://hagstofa.is/talnaefni/ibuar/mannfjoldi/.

[mgg370013-bib-0034] Þjóðskrá . 2021. “Registers Iceland.” Accessed June 5, 2023. https://www.skra.is/.

[mgg370013-bib-0035] Thornton, C. A. 2014. “Myotonic Dystrophy.” Neurologic Clinics 32, no. 3: 705–719. 10.1016/j.ncl.2014.04.011. 25037086 PMC4105852

[mgg370013-bib-0036] Tishkoff, S. A. , A. Goldman , F. Calafell , et al. 1998. “A Global Haplotype Analysis of the Myotonic Dystrophy Locus: Implications for the Evolution of Modern Humans and for the Origin of Myotonic Dystrophy Mutations.” American Journal of Human Genetics 62, no. 6: 1389–1402. 10.1086/301861. 9585589 PMC1377140

[mgg370013-bib-0037] Tulinius, H. 2011. “Multigenerational Information: The Example of the Icelandic Genealogy Database.” Methods in Molecular Biology (Clifton, N.J.) 675: 221–229. 10.1007/978-1-59745-423-0_11. 20949392

[mgg370013-bib-0038] Virtanen, P. , R. Gommers , T. E. Oliphant , et al. 2020. “SciPy 1.0: Fundamental Algorithms for Scientific Computing in Python.” Nature Methods 17, no. 3: 261–272. 10.1038/s41592-019-0686-2. 32015543 PMC7056644

[mgg370013-bib-0039] Vydra, D. G. , and A. Rayi . 2023. “Myotonic Dystrophy.” In StatPearls. Treasure Island, FL: StatPearls. https://pubmed.ncbi.nlm.nih.gov/32491378/. 32491378

[mgg370013-bib-0040] Yum, K. , E. T. Wang , and A. Kalsotra . 2017. “Myotonic Dystrophy: Disease Repeat Range, Penetrance, Age of Onset, and Relationship Between Repeat Size and Phenotypes.” Current Opinion in Genetics & Development 44: 30–37. 10.1016/j.gde.2017.01.007. 28213156 PMC5447468

